# A Densely Interconnected Genome-Wide Network of MicroRNAs and Oncogenic Pathways Revealed Using Gene Expression Signatures

**DOI:** 10.1371/journal.pgen.1002415

**Published:** 2011-12-15

**Authors:** Chia Huey Ooi, Hue Kian Oh, Hannah Zhu'Ai Wang, Angie Lay Keng Tan, Jeanie Wu, Minghui Lee, Sun Young Rha, Hyun Cheol Chung, David Marc Virshup, Patrick Tan

**Affiliations:** 1 Cancer and Stem Cell Biology Program, Duke-NUS Graduate Medical School, Singapore, Singapore; 2 Cellular and Molecular Research, National Cancer Centre, Singapore, Singapore; 3 Duke-NUS Genome Biology Facility, Duke-NUS Graduate Medical School, Singapore, Singapore; 4 Department of Internal Medicine, Yonsei Cancer Center, Yonsei University College of Medicine, Seoul, Republic of Korea; 5 Cancer Science Institute of Singapore, Yong Loo Lin School of Medicine, National University of Singapore, Centre for Life Sciences, Singapore, Singapore; 6 Genome Institute of Singapore, Singapore, Singapore; Stanford University Medical Center, United States of America

## Abstract

MicroRNAs (miRNAs) are important components of cellular signaling pathways, acting either as pathway regulators or pathway targets. Currently, only a limited number of miRNAs have been functionally linked to specific signaling pathways. Here, we explored if gene expression signatures could be used to represent miRNA activities and integrated with genomic signatures of oncogenic pathway activity to identify connections between miRNAs and oncogenic pathways on a high-throughput, genome-wide scale. Mapping >300 gene expression signatures to >700 primary tumor profiles, we constructed a genome-wide miRNA–pathway network predicting the associations of 276 human miRNAs to 26 oncogenic pathways. The miRNA–pathway network confirmed a host of previously reported miRNA/pathway associations and uncovered several novel associations that were subsequently experimentally validated. Globally, the miRNA–pathway network demonstrates a small-world, but not scale-free, organization characterized by multiple distinct, tightly knit modules each exhibiting a high density of connections. However, unlike genetic or metabolic networks typified by only a few highly connected nodes (“hubs”), most nodes in the miRNA–pathway network are highly connected. Sequence-based computational analysis confirmed that highly-interconnected miRNAs are likely to be regulated by common pathways to target similar sets of downstream genes, suggesting a pervasive and high level of functional redundancy among coexpressed miRNAs. We conclude that gene expression signatures can be used as surrogates of miRNA activity. Our strategy facilitates the task of discovering novel miRNA–pathway connections, since gene expression data for multiple normal and disease conditions are abundantly available.

## Author Summary

MicroRNAs (miRNAs) are naturally occurring small RNA molecules of ∼22 nucleotides that regulate gene expression. Recent studies have shown that miRNAs can behave as important components of cellular signaling pathways, as pathway regulators or pathway targets. Currently however, only a few miRNAs have been functionally linked to specific signaling pathways, raising the need for novel approaches to accelerate the identification of miRNA–pathway connections. Here, we show that gene expression signatures, previously used to reflect patterns of pathway activation, can also be used to represent miRNA activities. Using this approach, we constructed a genome-wide miRNA–pathway network predicting the associations of 276 human miRNAs to 26 oncogenic pathways. The miRNA–pathway network confirmed a host of previously reported miRNA/pathway associations and uncovered several novel associations that were subsequently experimentally validated. Besides being the first study to conceptually demonstrate that expression signatures can act as surrogates of miRNA activity, our study provides a large database of candidate pathway-modulating miRNAs, which researchers interested in a particular pathway (e.g. Ras, Myc) are likely to find useful. Moreover, because this approach solely employs gene expression, it is immediately applicable to the thousands of microarray data sets currently available in the public domain.

## Introduction

MicroRNAs (miRNAs) are naturally occurring small RNA molecules of ∼22 nucleotides that negatively regulate gene expression. Current models propose that miRNAs bind to complementary sequences in the 3′ untranslated regions (UTRs) of target mRNAs, causing either target mRNA degradation or reduced protein translation [1], [2]. miRNAs play important roles in cellular differentiation, proliferation, and apoptosis, and miRNA deregulation has been implicated in cancer [1]. Emerging evidence suggests that miRNAs can also play essential roles in canonical signaling pathways, acting either as regulators of pathway output or as important pathway targets [3], [4], [5]. For example, a recent study has identified the *miR-2355* cluster as a critical regulator of the TGF-β signaling pathway [6]. However, although hundreds of miRNAs have been discovered; to date only relatively few miRNAs have been linked to specific signaling pathways. Novel approaches are thus needed to accelerate the identification of miRNA–pathway connections.

Attempts have been made to identify miRNA–pathway relationships on a genome-wide scale [7], [8], [9]. However, most of these previous studies have typically relied on DNA sequence-based computational predictions, comparing lists of genes predicted to be miRNA targets against gene sets of pathway components and cellular functions (e.g. Biocarta and Gene Ontologies). While informative, studies relying primarily on miRNA target sequence predictions may suffer from the limitations of current-generation sequence-based prediction algorithms (e.g., TargetScanS, miRanda, and PITA) which have been shown to produce excessively large numbers of false positives among predicted miRNA target genes [10]. Studies purely based on computational DNA sequence predictions also rarely incorporate actual experimental transcriptomic information, and thus typically can neither determine if a particular miRNA is truly coexpressed with a target pathway component, nor with any other coexpressed miRNAs, in the same cell or tissue. Complementary methodologies are thus needed to explore the true biological diversity of miRNA–pathway relationships.

We, along with several others, have previously used gene expression signatures to predict the activity of oncogenic signaling pathways. In this approach, gene expression profiles of samples exhibiting activation or repression of a specific pathway are compared, producing a list of differentially-expressed genes as a surrogate of that pathway's activity. Once identified, this “pathway signature” can then be subsequently mapped onto independent samples from a wide diversity of disease conditions and tissues. Expression signatures of pathway activity have been used to define distinct subtypes of cancer ([11], uncover prevalently mutated cancer pathways [12], [13], and predict responses to targeted therapies [14]. However, although the concept of using gene expression signatures to predict signaling pathway activity is well-established, to our knowledge no study has investigated if similar signatures can also be used to predict patterns of miRNA activity.

In this study, we addressed this question and demonstrate that gene expression signatures can indeed be used to predict the activity status of specific miRNAs. By combining hundreds of expression signatures representing pathways and miRNAs, we created a miRNA–pathway network allowing the identification of miRNA–pathway connections on a high-throughput, genome wide scale. Notably, because our approach only requires gene expression information, it is readily applicable to the thousands of gene expression data sets currently available in the public domain.

## Results

### Gene Expression Signatures as Surrogates of miRNA Activity

**Figure 1:**
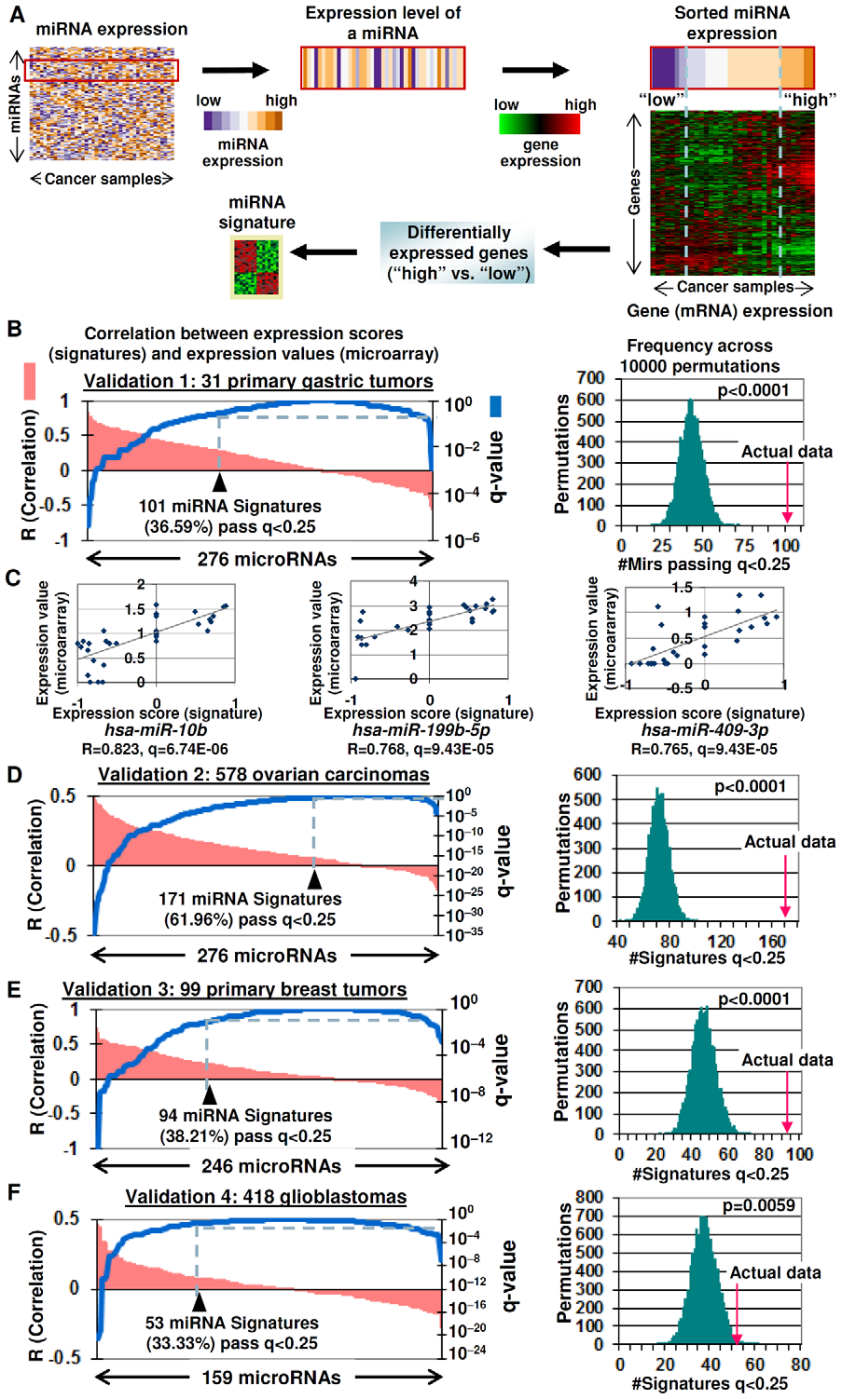
Gene expression signatures as surrogates of miRNA expression. A) Generating miRNA gene expression signatures. Differentially expressed genes are identified between samples expressing high and low miRNA levels (nominal p<0.0001). B) Validation of miRNA gene expression signatures (Validation Set #1:31 primary gastric tumors). (Left graph) Plot of Spearman correlations between miRNA gene expression scores and miRNA expression values (pink) and corresponding q-values (blue). The black triangle highlights the number of miRNA signatures passing the R>0, q<0.25 threshold. (Right graph) Distribution of miRNAs exhibiting positive correlations between miRNA gene expression scores and miRNA expression values (R>0 and q<0.25), after the miRNA labels were randomly shuffled across 10000 permutations. The red arrow represents the number of positively correlated miRNAs in the actual data. C) Examples of miRNAs in Validation set #1 showing high correlations between miRNA gene expression scores and miRNA expression values. All p-values were corrected for multiple hypotheses (shown as q-values). D-F) Validation of miRNA gene expression signatures in (D) Validation Set #2:578 ovarian carcinomas, (E) Validation Set #3:99 primary breast tumors, and (F) Validation Set #4:418 glioblastomas. For Validation Sets #3 and #4, the numbers of miRNAs analyzed were limited to miRNAs present in both the gastric-derived 276 miRNA signatures and miRNA expression data in the validation set.

We conceived a strategy for generating gene expression signatures representing miRNA activity (Figure 1A). First, a training dataset is established comprising biological samples for which both miRNA and mRNA gene expression profiles are known. Second, to build a gene expression signature for any given miRNA *X*, we chose biological samples where miRNA *X* was either overexpressed (“miRNA high” group; ≥ the 80th percentile of all samples) or underexpressed (“miRNA low” group; ≤20th percentile). Each miRNA was analyzed independently. Third, we used LIMMA (Linear Models for Microarray Data), a modified t-test incorporating the Benjamini Hochberg multiple hypotheses correction technique [15], to identify differentially expressed genes (mRNAs) between the “miRNA high” and the “miRNA low” groups (significance level p<0.0001). These genes form a candidate signature representing activity of that miRNA. Although this approach requires an initial training data set for which miRNA and mRNA are simultaneously available, this initial step needs only to be done once - once the gene expression signature representing the miRNA has been generated, this signature can then be subsequently mapped against any cohort comprising only mRNA expression information to infer miRNA activity in that cohort.

For clarity, we define three terms that will be used in this study. “miRNA gene expression signature” will refer to a gene expression based signature of miRNA activity. “miRNA gene expression score” will be used to represent the level of miRNA activity predicted by the miRNA gene expression signature, while the term “miRNA expression value” will refer to an actual experimentally determined level of miRNA expression (e.g., measured using an miRNA microarray).

We applied this approach on a training miRNA/mRNA expression dataset of 43 gastric cell lines where each sample had been profiled for expression of 799 miRNAs and 47,000 mRNA transcripts (see Methods). Of the 799 miRNAs, we were able to generate miRNA gene expression signatures for 516 miRNAs (64.6%) above the threshold level of significance (p<0.0001). The remaining 283 miRNAs were not analyzed further, as they were not associated with sufficient numbers of samples in either the “miRNA high” or “miRNA low” groups for meaningful statistical analysis. In this analysis, the minimum number of samples in either the “miRNA high” or “miRNA low” groups was mandated to be three, since this is the minimum number of samples required for meaningful LIMMA analysis. There was no upper limit. The 516 miRNA signatures were then subjected to a robustness test to select signatures passing a False Discovery Rate cutoff of <5% (see Methods, “Robustness of miRNA Gene Expression Signatures”). This operation resulted in a candidate set of 276 miRNA signatures. The complete gene sets and size distributions of the miRNA signatures are presented in Table S1 and Table S2.

To test the ability of the miRNA gene expression signatures to act as surrogates of miRNA expression, we used a previously-described signature mapping technique [11] (Methods and Text S1). Specifically, we mapped the 276 miRNA gene expression signatures onto an independent cohort of 31 primary gastric tumors for which both miRNA and mRNA profiles were available. In this validation analysis, a miRNA signature were deemed to be significantly correlated to actual miRNA expression if the signature was positively correlated to the miRNA (Spearman's correlation R>0) exceeding a threshold q-value of <0.25, where the q-value denotes the significance of the correlation (see Methods). Using this criterion, more than a third of the miRNA gene expression scores (101 out of 276 miRNA signatures, 36.59%) were positively correlated to their actual miRNA expression values across the tumors (Spearman's correlation R>0, q<0.25) (Figure 1B, left panel). Figure 1C shows some examples of miRNAs exhibiting good correlations between miRNA gene expression scores and actual miRNA expression values in this independent test set, such as *hsa-miR-10b*, *hsa-miR-199b-5p*, and *hsa-miR-409-3p*. To evaluate the likelihood that these correlations might have been obtained by chance, we performed a global permutation analysis where the miRNA labels were scrambled in the primary tumor test set 10,000 times, and the percentage of miRNAs exhibiting positive Spearman correlations (R>0, q<0.25) between miRNA gene expression scores and miRNA expression values were computed (see Methods). We found that the number of miRNAs showing positive correlations between miRNA expression values and miRNA expression scores in the actual dataset (101 miRNAs) was consistently greater than that obtainable in 10,000 randomly permuted data sets (i.e., p<0.0001, average number 42 per permutated data set) (Figure 1B, right panel) (Table S3). This result indicates that it is highly unlikely that the observed correlations between miRNA gene expression scores and miRNA expression values are obtained by chance alone.

To investigate the biological applicability of the gastric-derived miRNA signatures beyond gastric cancer, we then analyzed three additional independent validation sets for which gene expression and miRNA data were available (Figures 1D–1F). In a series of 578 ovarian cancers (http://tcga-data.nci.nih.gov/tcga/tcgaHome2.jsp), 61.96% (171 out of 276) of the gastric-derived miRNA signature expression scores were positively correlated to their actual miRNA expression values determined by a miRNA microarray (threshold R>0; q-value q<0.25). Again, this percentage was significantly greater compared to 10,000 randomly permuted data sets (ie p<0.0001) (Figure 1D and Figure S1). In a series of 99 breast tumors (GEO, GSE19783), 38.21% (94 of 246) of the miRNA signature expression scores were positively correlated to their actual miRNA expression values (p<0.0001) (Figure 1E). Finally, in a series of 418 glioblastomas (http://tcga-data.nci.nih.gov/tcga/tcgaHome2.jsp), 33.33% (53 of 159) of the miRNA expression scores were positively correlated to their actual miRNA expression values (p = 0.0059) (Figure 1F).

In a further comparison, we retrieved a signature based on genes differentially expressed in *hsa-miR-155*-transfected HEK-293 cells compared to control cells (p<0.0005; GSE9264) [17], used it to classify the two largest validation data sets (418 glioblastomas and 578 ovarian cancers), and compared the classification to one based on the gastric-derived *hsa-miR-155* miRNA expression scores. For both validation sets, there was either a highly significant or near-significant concordance between tumors assigned as “mir-155 positive” by the HEK293 signature and those tumors assigned as “mir-155 positive” by the gastric-derived hsa-miR-155 expression signature (p = 5.8×10−7 for glioblastoma and p = 0.06 for ovarian cancer; chi-square test) (Table S4). Taken collectively, these results support the notion that miRNA gene expression signatures can indeed recapitulate actual miRNA expression patterns in a variety of tissues.

### Gene Expression Signatures Identify miRNA–Pathway Connections

**Figure 2:**
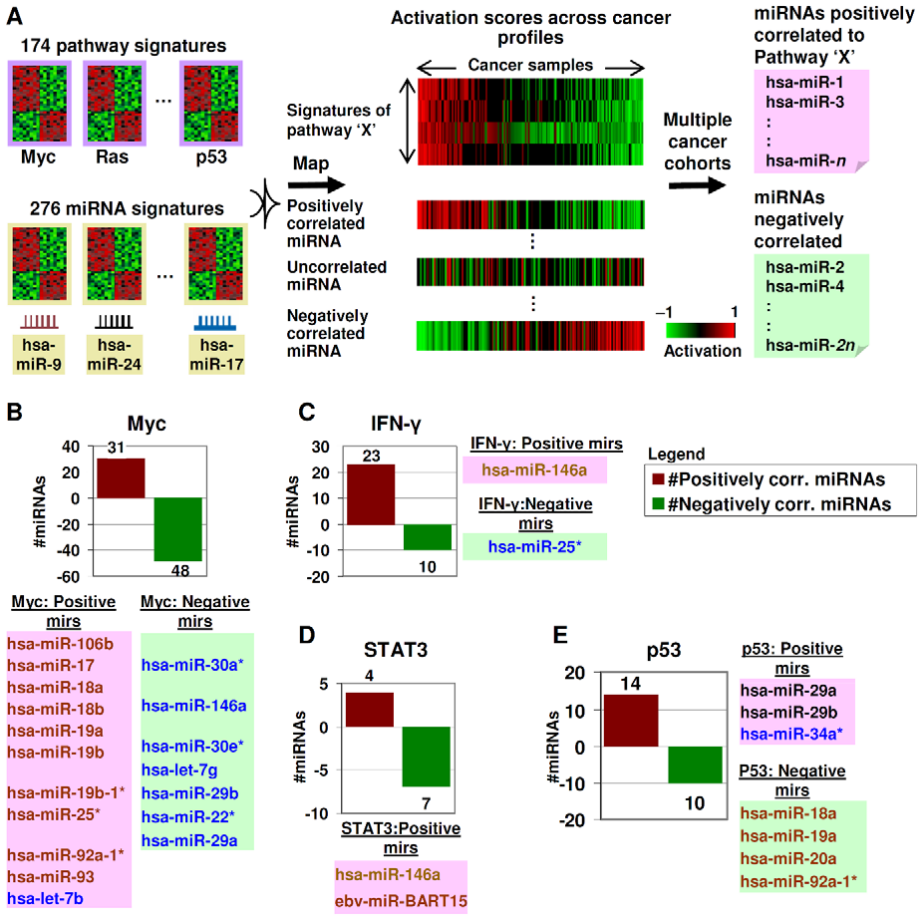
Identifying miRNA–pathway connections. A) Pathway and miRNA gene expression signatures are mapped onto gene expression profiles of samples. For every pathway, positively and negatively correlated miRNAs are identified. This process is repeated across multiple cohorts to identify robust connections. B) miRNA-Myc connections. Positively and negatively correlated miRNAs to Myc are shown in red and green. Correlated miRNAs previously reported as Myc-regulated are highlighted in brown (Myc-induced) and blue (Myc-repressed). C) miRNA-IFN-γ connections. Correlated miRNAs previously reported as connected to IFN-γ are highlighted. D) miRNA-STAT3 connections. Correlated miRNAs previously reported as connected to STAT3 are highlighted. E) miRNA-p53 connections. Correlated miRNAs previously reported as regulated by p53 are highlighted.

To identify miRNA–pathway connections, we mapped the 276 miRNA gene expression signatures against a series of cancer sample expression profiles. We then mapped onto the same profiles 174 pathway signatures representing 26 oncogenic pathways [11] (see Table S5 and Table S6 for the signatures and their pathway assignments) (Figure 2A). Integrating the two data sets, we computed correlations between the miRNA and pathway gene expression signatures, identifying miRNAs either positively or negatively correlated to the various pathways based on a preset significance threshold (see Methods for details). To focus on identifying robust miRNA–pathway connections, we repeated the miRNA and pathway signature mapping across five independent cancer cohorts – i) a panel of 39 gastric cancer cell lines, ii) a cohort of 200 primary gastric tumors, iii) a second cohort of 70 primary gastric tumors, iv) a cohort of 189 primary breast tumors, and v) another cohort of 286 primary breast tumors. We only retained those miRNA–pathway associations that a) did not show any contradictions across the cohorts, and b) also existed in the cell line cohort to facilitate subsequent *in vitro* validation studies (see below). There was a wide range in the numbers of miRNAs correlated to any particular pathway (5–262 miRNAs per pathway, mean ∼151 miRNAs, Table S7). Interestingly, the reproducibility of microRNA-pathway associations did not appear to be significantly influenced by the particular tissue type of the cancer cohorts (Table S8), although we emphasize that this certainly does not rule out the possibility that tissue-specific miRNA effects may, and are indeed likely, to also exist (see Discussion). A complete list of miRNA–pathway associations is available in Table S9, and Table S10 provides a list of the top miRNAs associated with the highest number of pathways.

We surveyed the identified miRNA–pathway interactions in the context of several specific pathways. Supporting the biological relevance of the predicted miRNA–pathway connections, we observed several previously reported and experimentally validated miRNA–pathway associations. A few examples are now presented:

#### Myc signaling

For the Myc pathway, sufficiently large numbers of Myc-associated miRNAs are known to enable statistical testing of the concordance between the miRNA–pathway connections predicted by our approach to those identified by previous studies [3], [4], . Of 10 miRNAs previously shown to be induced by Myc, all 10 miRNAs were also predicted by our approach to be positively correlated to Myc; while conversely of 8 miRNAs previously shown to be repressed by Myc, 7 were also predicted by our approach to be negatively correlated to Myc (Figure 2B, p = 0.000251, Fisher's exact test, Table S11).

#### IFN-signaling

IFN-γ protein secretion has been reported to be increased by *hsa-miR-146a* [17], while *hsa-miR-25* has been reported to be repressed in airway smooth muscle cells treated with cytokines IL-1β, TNF-α, and IFN-γ [18]. Using our approach, *hsa-miR-146a* and *hsa-miR-25* were predicted to be positively and negatively associated with IFN-signaling, respectively (Figure 2C).

#### STAT signaling

Two miRNAs (*hsa-miR-146a* and a EBV-related miRNA) were predicted by our approach to be positively associated with STAT signaling. *hsa-miR-146a* (Figure 2D, labeled brown) has been reported to modulate STAT3 signaling [19]. The connection of *hsa-miR-146a* to both the IFN and STAT pathways is not unexpected, as *hsa-miR-146a* has been linked to inflammatory/immune responses involving both pathways [20]. The correlation of an EBV miRNA (Figure 2D, labeled red) to the STAT3 pathway may be due to the reported effect of EBV infection on STAT3 activation [21].

#### P53 signaling

Our approach identified *hsa-mir-29a*, *hsa-mir-29b*, and hsa-mir-34a* as being positively correlated to p53 signaling. Supporting this interaction, miRNAs of the *mir-29* family have been previously reported to activate p53 [5], and hsa-miR-34a* is the minor sequence of the *miR-34a* stem-loop which produces *hsa-miR-34*, a p53 inducible miRNA [22]. Likewise, we identified hsa-mir-18a, hsa-mir-19a, hsa-mir-20a and hsa-mir-92a-1* as being negatively correlated to p53 signaling. These four miRNAs are part of the *miR-17-92* cluster, which is repressed by p53 [23] (Figure 2E).

### Gene Expression Signatures Identify Novel miRNA Modulators of Wnt Signaling

**Figure 3:**
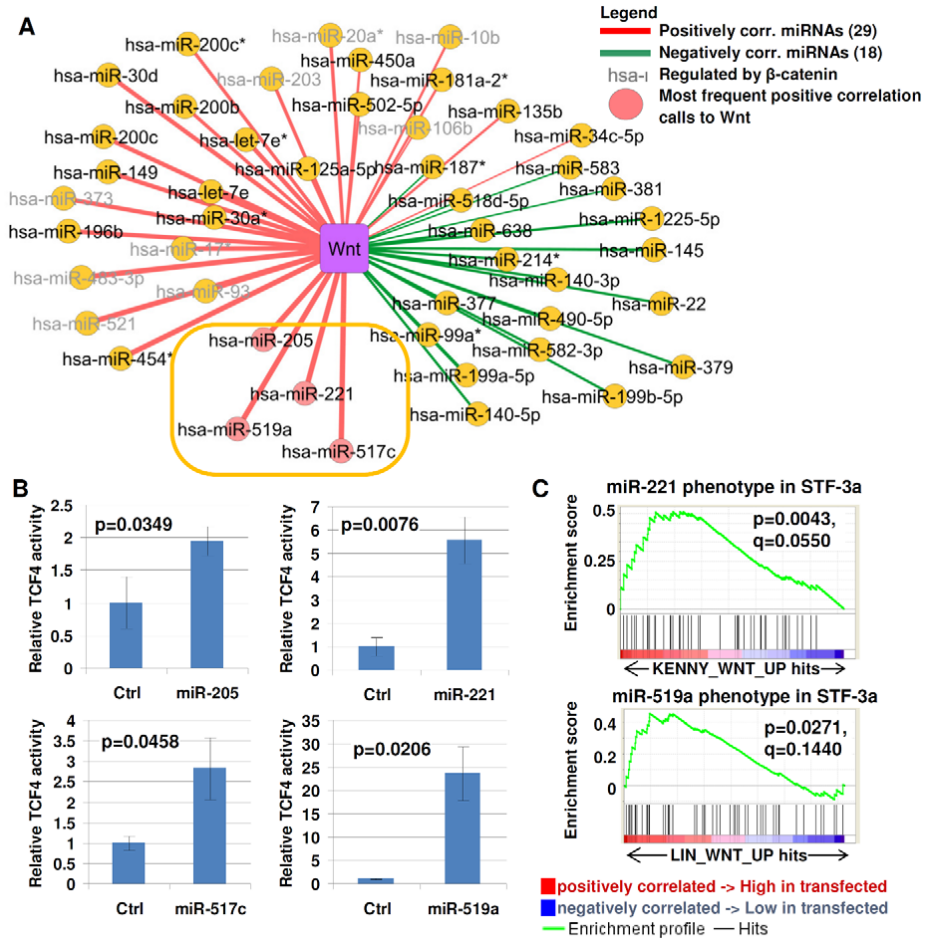
Identifying miRNA–Wnt pathway connections. A) Wnt correlated miRNA. Yellow nodes indicate miRNAs, purple nodes indicate pathways. Color of lines indicate the correlation type (red = positive, green = negative). Thickness of lines indicates frequency of correlation calls across 5 cohorts. miRNAs with the highest frequency of positive correlation calls to the Wnt pathway are marked with pink circles. miRNAs regulated by β-catenin are labeled in grey. The gold oval highlights the miRNAs selected for functional testing. B) TCF/LEF transcriptional acitivity in STF-3a cells transfected with candidate miRNAs. Non-targeting sequences were used as a negative control. (y-axis) TOP-flash reporter activity was measured in RLU (relative light units). C) Geneset enrichment analysis of miRNA-tranfected cells. Top panel, enrichment plot for the Wnt activation signature (KENNY_WNT_UP) in miR-221 overexpressing cells. Bottom panel, enrichment plot for the Wnt activation signature LIN_WNT_UP in miR-519a overexpressing cells. The nominal p-value estimates the statistical significance of the enrichment score for a single geneset. The q-value is the FDR (false discovery rate), adjusted for gene set size and multiple hypotheses testing among MSigDB (C2 collection, Release 2.5).

We then explored if our approach could identify novel miRNA–pathway associations not previously reported in the literature. For this purpose, we focused on the Wnt signaling pathway, since few miRNAs are known to be associated with Wnt signaling. Among the 276 miRNAs analyzed, we identified 29 and 18 miRNAs positively and negatively correlated to Wnt signaling, respectively. To enrich for miRNAs acting as upstream modulators of Wnt signaling rather than downstream targets, we considered from this set only those miRNAs that were a) consistently positively associated with the Wnt pathway in all the cohorts analyzed (Figure 3A), and b) whose expression was not affected by a β-catenin knockdown experiment in AGS gastric cancer cells (see Methods and Table S12). Using these criteria, 4 miRNAs, *hsa-miR-205*, *hsa-miR-221*, *hsa-miR-517c*, and *hsa-miR-519a* (inside orange rectangle, Figure 3A) were nominated as candidate regulators of Wnt signaling.

To assess the effects of these four miRNAs on Wnt pathway activity, we transfected pri-microRNA sequences of *hsa-miR-205*, *hsa-miR-221*, *hsa-miR-517c*, and *hsa-miR-519a* into STF-3a cells, which are HEK293 embryonic kidney cells constitutively expressing a TOP-Flash reporter plasmid and overexpressing WNT-3a [24]. TOP-Flash is a luciferase expressing plasmid containing multimerized TCF/LEF binding sites, a standard reporter assay for determining Wnt/β-catenin activity [25]. Compared to control transfected cells, significantly higher TOP-Flash transcriptional activities were observed in STF-3a cells transfected with *hsa-miR-205*, *hsa-miR-221*, *hsa-miR-517c*, or *hsa-miR-519a*, ranging from 2- to 25-fold induction (Figure 3B). To extend our analysis beyond the artificial TOP-Flash reporter, we generated gene expression profiles of STF-3a cells transfected with *pri-hsa-miR-221*, and explored if endogenous Wnt target genes might be regulated by this miRNA. Geneset enrichment analysis (GSEA, [26]) of genes up-regulated in *hsa-mir-221* transfected STF-3a cells revealed significant enrichment of the geneset KENNY_WNT_UP (top panel, Figure 3C), corresponding to genes up-regulated in HC11 mammary epithelial cells by expression of constitutively active CTNNB1, a key component of the Wnt pathway [27]. Similarly, GSEA of *hsa-miR-519a*-transfected STF-3a cells showed significant enrichment of the geneset LIN_WNT_UP, comprising Wnt target genes identified by expression of the Wnt antagonist *APC* in APC-deficient SW480 colon cancer cells [28] (bottom panel, Figure 3C). These two Wnt-related gene sets (KENNY_WNT_UP and LIN_WNT_UP) were noted because they exhibited the highest GSEA enrichment scores among all Wnt-related genesets in the MSigDB C2 collection for *hsa-mir-221* and *hsa-miR-519a* respectively (Tables S13 and S14). The observation that *hsa-miR-221* and *hsa-miR-519a* transfections can activate two different Wnt-related genesets suggests that these two miRNAs may contribute to distinct Wnt-related regulatory cascades. No increases in TCF/LEF transcriptional activity were observed when the miRNA transfections were performed on STF cells not expressing WNT-3a (data not shown). These results suggest these miRNAs may modulate Wnt activity, but can only do so in the presence of an active Wnt ligand. Interestingly, while not achieving significance, several genes upregulated in the gastric-derived *mir-221* signature were also upregulated in expression profiles of *hsa-mir-221* transfected STF-3a cells (positive Normalized Enrichment Score (NES) = 0.84), and several genes downregulated in the gastric-derived *mir-221* signature were also downregulated in the hsa-mir-221 transfected STF-3a cells (negative Normalized Enrichment Score (NES) =  −1.652) (Figure S2). For mir-519a, we again found that several genes upregulated in the gastric-derived mir-519a signature were also found to be upregulated in hsa-mir-221 transfected STF-3a cells (positive Normalized Enrichment Score (NES) = 1.091). Only one gene was downregulated in the *hsa-mir-519a* signature, which is insufficient for GSEA analysis. These results may suggest that certain genes belonging to a miRNA signature may themselves be directly or indirectly regulated by that miRNA.

### The miRNA–Pathway Network Reveals a High Level of Functional Redundancy

**Figure 4:**
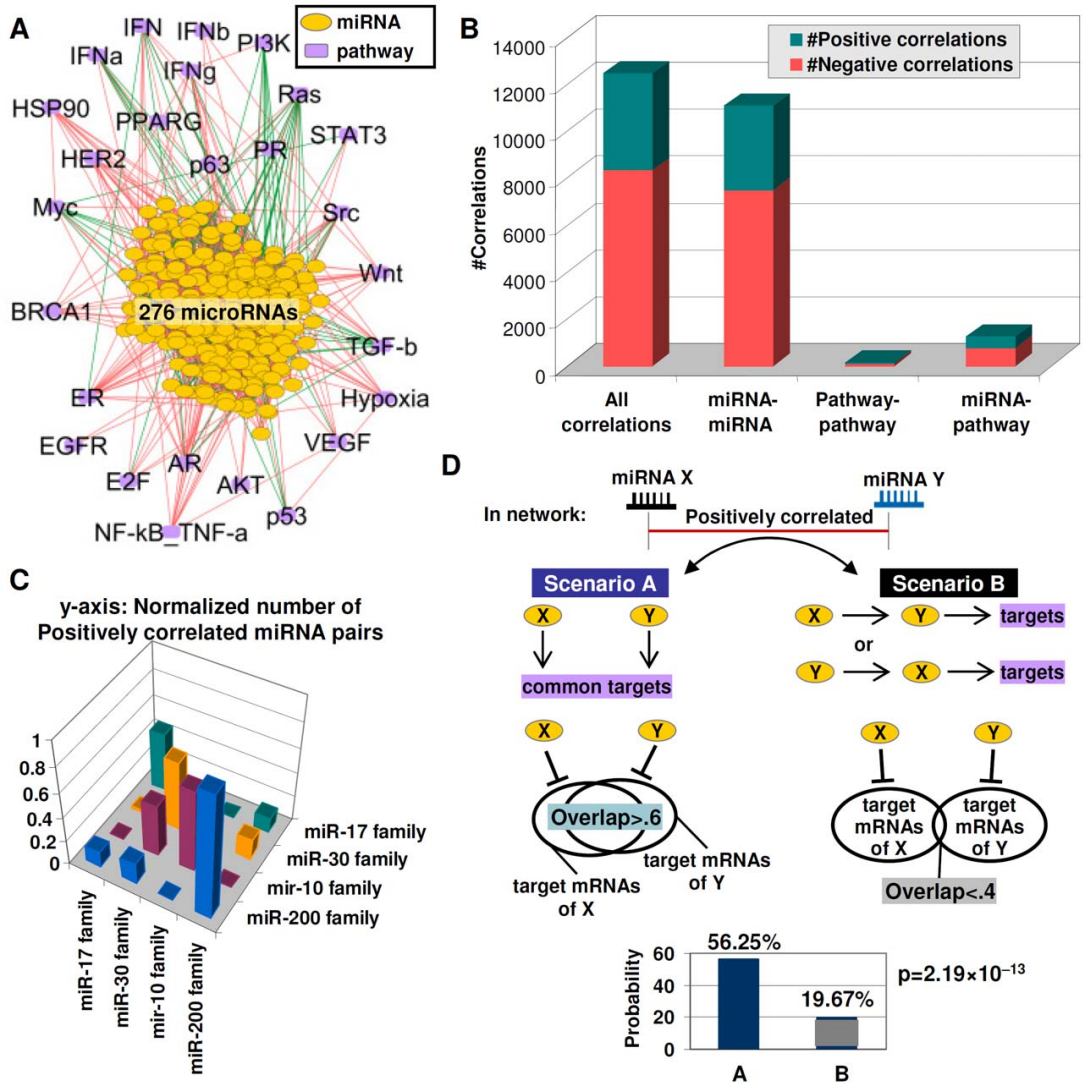
Functional redundancy in the miRNA–pathway network. A) The miRNA–pathway network. Lines represent significant correlations across 276 miRNAs and 26 pathways (red = positive, green = negative). Yellow nodes are miRNAs and purple nodes are pathways. B) Number of positive and negative correlations between different node types (miRNA-miRNA, pathway-pathway, miRNA–pathway). C) Frequency of positively correlated miRNA pairs within and between miRNA families. A taller column indicates a higher frequency of positive miRNA-miRNA correlations. miRNA pairs from the same family show a higher frequency of correlations compared to miRNA pairs between different families. D) Possible scenarios explaining positive correlations between two miRNAs. Scenario A (left) – miRNAs act independently but have common downstream effects (redundancy); Scenario B (right) – one miRNA directly regulates the second miRNA. (bottom) miRNAs with significant overlaps in target mRNAs (overlap ratio >0.6) are more likely to be positively correlated compared to miRNAs with small target mRNA overlaps (overlap ratio <0.4). y-axis: probability of positive correlation. x-axis: miRNA pairs with overlap ratios of >0.6 or <0.4. p-values were computed using chi-square test.

The availability of an extensive catalog of miRNA–pathway connections provided us with the opportunity to analyze the global properties of the miRNA–pathway network. Integrating gene expression signatures from 276 miRNAs passing the signature robustness test (see Methods) and 174 pathway signatures representing 26 oncogenic pathways, we constructed a miRNA–pathway network of 302 nodes and 12442 edges (Figure 4A, see Methods for network construction). Here, ‘node’ refers to either a pathway or miRNA signature, and edges refer to significant correlations between nodes, which can be either positive (“positive edges”) or negative (“negative edges”).

We found that the miRNA–pathway network is dominated by positive edges (8327 positive vs. 4115 negative) (Figure 4B), similar to previously reported gene co-expression networks [29]. miRNA-miRNA connections form the bulk of edges in the network, an expected result given the higher number of miRNA nodes (Figure 4B). Formally, positive edges connecting two miRNAs X and Y can reflect two different scenarios. In Scenario A, miRNAs X and Y act independently from one another to exert similar downstream effects i.e., X and Y exhibit “redundancy”. In Scenario B, miRNA X may regulate miRNA Y, with Y proceeding to exert the actual downstream effect, or vice versa (Figure 4D). We sought to determine which of these scenarios might characterize the miRNA-miRNA edges in the network. First, we tested if positive miRNA-miRNA edges in the network might be selectively enriched in members from the same miRNA family, since same family-miRNAs are known to exhibit redundant or partially redundant functions [30]. Testing four different miRNA families (*miR-200* family; *mir-17* family; *miR-30* family; *miR-10* family; Table S15) (Figure 4C), we found that that a miRNA was >5 times more likely to be positively correlated to another miRNA from the same family, rather than a miRNA from a different family (67.02% vs. 10.90%, p = 4.76×10^−29^, Table S16), supporting the notion that miRNA-miRNA edges in the network are likely to identify functionally redundant miRNAs.

We extended our analysis from same-family miRNAs to all miRNAs in the network. Since functionally-redundant miRNAs are likely to share significant overlaps of predicted target mRNA sequences, we used four sequence-based prediction databases (MiRanda, PicTar, TarScan, and PITA; Table S17) to identify predicted target mRNAs for 215 miRNAs in the network (78%) (Figure 4D). We found that miRNAs with significant overlaps in target mRNAs (overlap ratio>0.6, see Methods) were >2 times more likely to be connected via positive miRNA-miRNA edges compared to miRNAs with small target mRNA overlaps (56.25% vs. 19.67%, p = 2.19×10^−13^, Figure 4D, Table S18). These results suggest that positive miRNA-miRNA edges in the network are likely to represent miRNA pairs with a high degree of functional redundancy, as evidenced by their targeting similar sets of downstream genes.

### Oncogenic Signaling Pathways Frequently Target miRNAs Exerting Similar Downstream Effects

**Figure 5:**
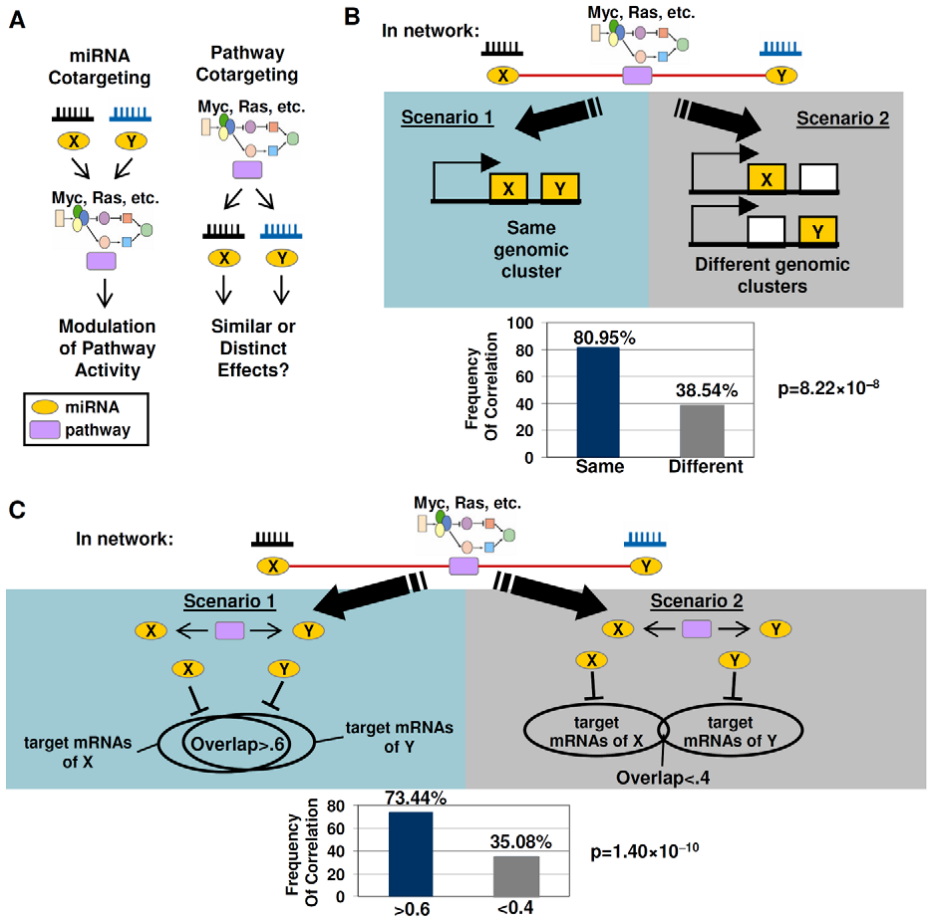
Cotargeting of miRNAs by oncogenic signaling pathways. A) Models of miRNA cotargeting and pathway cotargeting. (left) In miRNA co-targeting, multiple independently acting miRNAs can target the same pathway to modulate pathway activity (right) In pathway cotargeting, a pathway acts to target multiple independent miRNAs, which exert either similar or distinct downstream effects. B) miRNAs jointly correlated to a common pathway tend to be expressed from the same genomic cluster. In Scenario 1, a miRNA pair regulating a common pathway is associated with the same genomic location. In Scenario 2, that miRNA pair occupies different locations.(bottom graph) miRNAs connected to the same pathway are likely to be transcribed from the same genomic cluster rather than from different genomic clusters. C) miRNAs jointly correlated to a common pathway tend to exhibit significant overlaps in target mRNAs. Scenario 1 – miRNA pairs jointly correlated to the same pathway tend to exert similar downstream effects ie high overlap of target mRNAs (overlap ratio >0.6). Scenario 2 – miRNAs pairs jointly correlated to the same pathway tend to exert distinct effects (overlap ratio <0.4). (bottom graph) miRNAs with significant overlaps of target mRNAs (overlap ratio >0.6) are more likely to be jointly connected to the same pathway. p-values were computed from a chi-square test.

Previous studies have proposed that signaling pathways are frequently targeted by multiple independent miRNAs (‘miRNA cotargeting’; [7] (Figure 5A, left). The availability of the miRNA–pathway network allowed us to investigate the reciprocal possibility – that oncogenic signaling pathways might also co-ordinately regulate several miRNAs with similar downstream effects, which we refer to as “pathway cotargeting” (Figure 5A, right). To identify candidate miRNAs co-targeted by the same pathway, we nominated pairs of miRNA where both members were jointly positively or negatively correlated to the same common pathway. Of 262 miRNAs in the network significantly correlated with at least one pathway, 8020 miRNA pairs (out of >20,000 possible pairs) were candidates for pathway cotargeting, including *hsa-miR-497* and *hsa-miR-503* (ER, Ras, and TGF-β), *hsa-miR-20a* and *hsa-miR-372* (EGFR and p53), and *hsa-miR-192* and *hsa-miR-215* (BRCA1 and HER2).

Our analysis suggests that miRNA pairs jointly correlated to the same pathway are likely to represent miRNAs commonly regulated by the pathway. Specifically, we found that miRNA pairs jointly correlated to the same pathway exhibited a strong preference to be expressed from the same chromosomal region, suggesting that they may possess common upstream regulators. Examining genomic clusters of commonly transcribed miRNAs [31], we found that miRNA pairs located in the same genomic cluster were twice as likely to be jointly correlated to the same pathway compared to miRNA pairs transcribed from different genomic clusters (80.95% vs. 38.54%, p = 8.22×10^−8^, chi-square test) (Figure 5B, Table S19). For example, *hsa-miR-18a* and *hsa-miR-19a*, which are transcribed at a miRNA cluster at 13q31.3, were jointly correlated to EGFR, IFN-γ, Myc, and p53. Indeed, these miRNAs have also been independently shown to be co-regulated by Myc signalling [16]. As another example, *hsa-miR-154* and *hsa-miR-377* located at 14q32.31 were jointly correlated to Myc, TGF-β, and VEGF pathways. The frequent occurrence of genomic co-localization (thereby assuming common upstream regulation) within miRNA pairs jointly correlated to the same pathway makes it likely that these miRNA pairs are co-regulated by the same pathway.

miRNAs exhibiting joint correlations with common pathways were also highly enriched in miRNA pairs exhibiting significant overlaps in predicted target mRNAs (overlap ratio>0.6) relative to miRNA pairs with small target mRNA overlaps (73.44% vs 35.08%, p = 1.4×10^−10^) (Figure 5C, Table S20a). This result suggests that miRNAs jointly correlated to a pathway tend to exert similar downstream effects. The high overlaps in predicted target mRNAs are unlikely to be explained by high levels of sequence similarity between miRNA pairs, since a control analysis using only miRNAs with pairwise sequence similarity scores of less than -5 (see Methods) yielded similar results (67.86% vs. 35.54%, p = 0.000357, Table S20b). Notably, some of those downstream effects may involve feedback activation or inhibition of the pathway itself.

### The miRNA–Pathway Network Exhibits a Small-World Modular Organization and Is Densely Interconnected

Finally, we expanded our analysis of the miRNA–pathway network to consider its global topological and network features. The term “small-world network” has been previously used to refer to a network organization that is highly clustered with small path lengths (average lengths of the shortest path connecting any two nodes). Examples of previously known small-world networks are neural circuits of the vertebrate brain [32], the power grid of the western United States, and the collaboration graph of film actors [33]. Our topological analysis suggests that the miRNA–pathway network is also likely to exhibit a small-world organization. Comparing topological features of the miRNA–pathway network to an equivalent random graph with the same number of nodes and degree, we found that the miRNA–pathway network fulfils two key prerequisites for a small-world network [33], referred to as *L_s_ > =  L_r_* and that *C_s_*>>*C_r_*. First, the characteristic path length of the miRNA–pathway network, *L*_microRNA−path_, is 1.7822, which is almost equal to, but greater than the characteristic path length for an equivalent random graph network, *L*_rand_ = 1.7241±0.0008 (mean±std from 1000 permutations, see Methods). Second, the clustering coefficient of the miRNA–pathway network, *C*_microRNA−path_ = 0.6351, is more than twice the clustering coefficient of the equivalent random graph network, *C*_rand_ = 0.2705±0.0013. For networks with small node numbers of nodes (∼200–3000, miRNA–pathway network: 302 nodes), the observation that *C*_microRNA−path_>*C*_rand_ is sufficient to demonstrate small-world properties [34].

**Figure 6:**
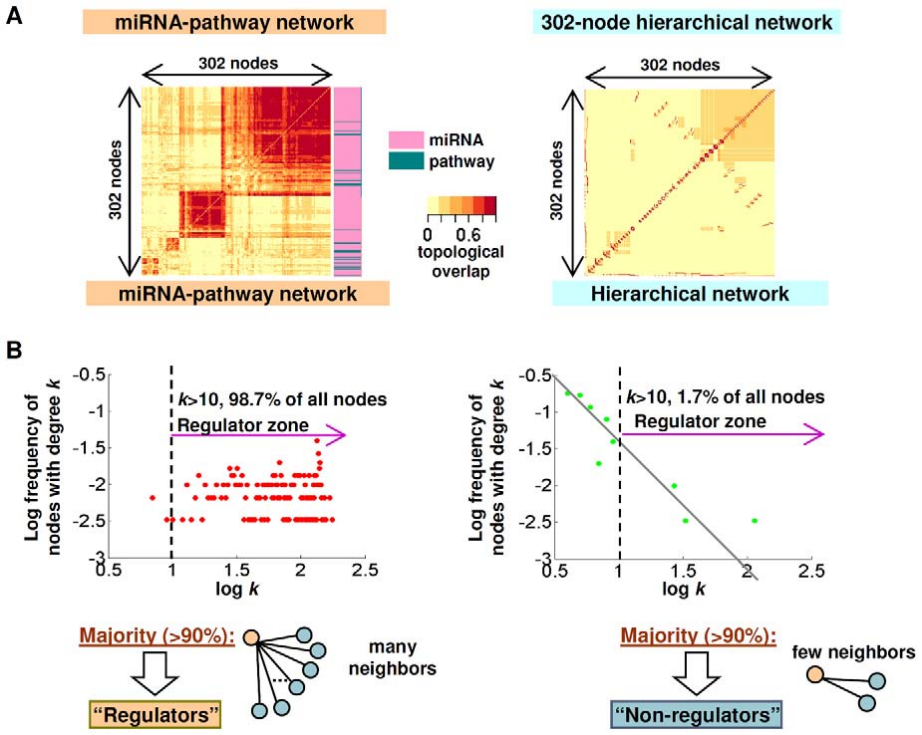
miRNA–pathway network topology. (A) Topological overlap matrix of the miRNA–pathway network (left) and a simulated network (right) of the same size but with a hierarchical, scale-free and modular architecture typical of gene regulatory and metabolic networks. Each row and column corresponds to a node. Rows and columns were ordered using unsupervised hierarchical clustering, Nodes that have large topological overlap are placed close together, resulting in modules of nodes. miRNAs and pathways nodes are indicated by red or green bars, respectively. (B) Degree distribution plots of the miRNA–pathway network (left) and a simulated network (right) of the same size but with a hierarchical, scale-free and modular architecture typical of gene regulatory and metabolic networks. y-axis: log frequency of nodes having the degree *k*, x-axis: log *k*.

This result also indicates that the miRNA–pathway network is more than twice as modular compared to an equivalent random graph, since the clustering coefficient *C_s_* is a measure of potential network modularity. Analyzing the miRNA–pathway network presented in Figure 4A, we found that the miRNA–pathway network is coherently structured in a strongly modular manner, with at least two major modules and two smaller modules (Figure 6A, left panel). In comparison, a simulated hierarchical network of the same size exhibited a higher number of modules (>20) that were distinctly smaller compared to the miRNA–pathway modules (Figure 6B, right panel). An even mix of miRNAs and pathways were localized to each module (Figure 6A, left panel).

Besides being small world, genetic and metabolic networks such as protein-protein interaction and yeast transcriptional or gene regulatory networks have also been shown to be “scale-free”, where the majority of connections are dominated by a small number of nodes (“hubs”) [35], [36]. To ask if the miRNA–pathway network might obey a scale-free character, we analyzed the degree distribution plot of the miRNA–pathway network, which characterizes the probability of a node having a certain number of neighbors. Strikingly, instead of obeying a power law, most of the connections in the miRNA–pathway network are of the many-to-many type (Figure 6B, left panel). Specifically, >98% of nodes in the miRNA–pathway network have more than 10 neighbors (Figure 6B, left panel), compared to only 1.7% in a simulated network typical of gene regulatory and metabolic networks (see Methods) (Figure 6B, right panel). This result suggests that the miRNA–pathway network is not scale-free. This result may be related to the observation that the miRNA–pathway network comprises mostly miRNAs that can regulate multiple pathways, and which are in turn regulated by multiple pathways. In other words, the miRNA–pathway network is primarily comprised of regulators. This observation is further pursued in the Discussion.

## Discussion

In this study, we have described a general approach for generating gene expression signatures which can be used to infer patterns of miRNA expression. Validation of this approach was demonstrated in both a training set and also an independent test set of gastric cancer samples. The member genes in the miRNA gene expression signature, while comprising genes transcriptionally altered as a consequence of miRNA activity, may not necessarily be direct miRNA target genes. This feature distinguishes our study from previous studies using sequence-predicted miRNA target genes to annotate miRNA functions. To our knowledge, our study is the first to demonstrate the ability of gene expression signatures to act as surrogates of miRNA activity. While the current analysis is limited to 276 miRNA signatures passing various quality and significance threshold cutoffs, applying this strategy to larger and more generalized training sets will undoubtedly identify more miRNA signatures. The current work should thus be regarded a proof-of-concept on the feasibility of gene expression signatures as surrogates of miRNA expression.

Using gene expression signatures to predict miRNA activity may address two major limitations currently facing miRNA–pathway discovery efforts – cost and scalability. Currently, most available experimental platforms (e.g., microarrays, deep sequencing) require the use of separate analytical assays to generate miRNA and mRNA information for a single sample (e.g., different microrarrays, or different RNA isolation techniques), increasing cost, time, and effort. Using gene expression signatures, it may be possible to analyze both miRNA and pathway activity patterns using a single common platform of gene expression. Moreover, because only gene expression information is required once the miRNA signature is known, any sample cohort for which gene expression (mRNA) data is available can be analyzed, without the requirement for companion miRNA data. This strategy thus opens up the availability of the thousands of publicly available microarray data sets for the discovery of new miRNA–pathway connections. Notably, we found that many of the miRNA signatures could recapitulate patterns of actual miRNA expression in a variety of different tumor types. This may not be too surprising, as it is conceptually similar to studies where gene expression signatures linked to pathways or drugs have been shown to exhibit broad applicability even in tissues distinct from those where the original signatures were derived (eg [14], [37]). However, we emphasize that our study does not rule out the possibility that miRNAs may exert tissue-specific effects.

One immediately useful application of miRNA gene expression signatures lies in identifying novel miRNAs linked to canonical signalling pathways. Using the miRNA–pathway network constructed in this study, we confirmed a host of previously reported miRNA pathway interactions, and identified four miRNAs as new candidate Wnt modulators (*hsa-miR-205*, *hsa-miR-221*, *hsa-miR-517c*, *hsa-miR-519a*). Experimental evidence supporting that these miRNAs are indeed Wnt regulators was also provided using cell line transfections, reporter assays, and gene expression profiling. Our study thus provides a large resource of potential pathway-modulating miRNAs for a variety of pathways which can be further tested by researchers. The information provided by this study is unlikely to be duplicated by other studies attempting to relate specific miRNAs to pathways and processes, as these previous studies have primarily relied on sequence-based miRNA target predictions, which have high false positive rates [10] and a general lack of tissue context – i.e., sequence-matches between a miRNA and a collection of mRNAs does not guarantee that the miRNA is indeed coexpressed with the target mRNA in the same cell type or tissue.

Besides miRNA interactions with individual pathways, our work reveals that co-expressed miRNAs are likely to exhibit a high degree of functional redundancy in targeting similar sets of downstream genes, and that signalling pathways may frequently cotarget multiple independent miRNAs with similar downstream effects. This observation extends previous studies [7] reporting the widespread existence of multiple pairs of miRNAs which target common genesets. Our observation that pathways frequently cotarget multiple miRNAs provides further evidence that miRNAs rarely act singly and almost always act in combinations to modulate cellular behaviour. The role of miRNAs as broad modulators may also explains the selection pressure for functional redundancies [30].

The functional role of miRNAs as broad modulators of cellular activities, rather than activators or repressors of specific genes, also explains the large modularity and non-scale-free attributes of the miRNA–pathway network, revealed by global topological analysis [1], [2]. The non-scale-free nature of the network is also likely explained by the membership of the network itself. Compared to the membership of typical scale-free genetic networks such as gene regulatory networks (GRNs) comprising a few master regulators and downstream effectors, the miRNA–pathway network is comprised entirely of regulators (miRNAs and pathways). An implication of the small-world but non-scale-free architecture is greater resilience to targeted “hits” than scale-free networks. Scale-free networks are resilient to randomly placed damage or failure, but susceptible to targeted attacks on the hubs (the few highly connected nodes), since such hits would remove a disproportionate amount of the links in the network [38]. The oncogenic miRNA–pathway network, with its small-world but non-scale-free architecture, does not rely on a few highly connected hubs, but spreads its “risk” across the many interconnected nodes (especially miRNAs). The implications of this finding on attempts to perturb cell function using miRNAs deserve further study.

In conclusion, our finding that gene expression signatures can capture miRNA activity is in general agreement with proposals that many cellular perturbations (e.g., responses to extracellular ligands, disease states, gene mutations) are likely to cause transcriptomic changes, and that these perturbations can be captured using gene expression signatures. Because functionally significant perturbations are certainly not limited to miRNAs and pathways alone, but can also include other genetic factors (SNPs, copy number variations, and mutation status) and epigenetic factors (e.g., DNA methylation and histone modification), there is in principle no reason why similar strategies could not be used to represent these other factors as well. Using gene expression signatures as a “common currency”, it may thus be possible to integrate multiple types of cellular perturbations into a common network, as we have done for miRNAs and pathways in this study. This may prove a powerful approach to identify functionally relevant relationships across a host of molecular levels that ultimately constitute the disease regulatory landscape.

## Materials and Methods

### Cell Lines and Primary Clinical Specimens

GC cell lines AGS, Kato III, SNU1, SNU5, SNU16, NCI-N87, and Hs746T were obtained from the American Type Culture Collection. AZ521, Ist1, TMK1, MKN1, MKN7, MKN28, MKN45, MKN74, Fu97, and IM95 cells were obtained from the Japanese Collection of Research Bioresources/Japan Health Science Research Resource Bank and cultured as recommended. SCH cells were a gift from Yoshiaki Ito (Institute of Molecular and Cell Biology, Singapore) and were grown in RPMI media. YCC1, YCC3, YCC6, YCC7, YCC10, YCC11, and YCC16 cells were a gift from Sun-Young Rha (Yonsei Cancer Center, South Korea) and were grown in MEM supplemented with 10% fetal bovine serum (FBS), 100 units/mL penicillin, 100 units/mL streptomycin, and 2 mmol/L L-glutamine (Invitrogen). In total, 39 unique GC cell lines, 2 fibroblast cell lines, and 1 normal gastric epithelial cell line (HFE145) were profiled. Wnt activity was assessed using STF-3a cells, which are HEK293 cells engineered to stably express WNT3A and a SuperTopFlash (STF) reporter gene [24]. Primary gastric tumors were obtained from the Singhealth Tissue Repository, an institutional resource of National Cancer Centre of Singapore and Singapore General Hospital. All patient samples were obtained with informed patient consent and approvals from Institutional Review Boards and Ethics Committees.

### miRNA and Gene Expression Profiling

Gastric cell lines and primary tumors were profiled using Human miRNA (V2) Microarrays (Agilent) and Affymetrix Human Genome U133 plus Genechips (HG-U133 Plus 2.0, Affymetrix). Where multiple probes existed for a unique miRNA, we took the probe providing the highest variance across samples as representative of that miRNA. The miRNA and gene expression data used in this manuscript can be accessed from the Gene Expression Omnibus (GEO) under accession numbers GSE22183 (miRNA, cell lines), GSE23739 (miRNA, primary tumors), GSE15459 (mRNA, primary tumors), GSE2990 (mRNA, 189 breast tumors), and GSE2034 (mRNA, 286 breast tumors). For independent validations, we used a breast cancer data set (GSE19783, mRNA and miRNA datasets analyzed using series matrices provided at GEO), and the TCGA ovarian cancer and glioblastoma data sets (http://tcga-data.nci.nih.gov/tcga/tcgaHome2.jsp; Ovarian: Level 2 datasets (mRNA (U133A) and miRNA (Agilent V2); Glioblastoma: Level 2 datasets (mRNA (U133A) and miRNA (Agilent V1)). Only samples having both mRNA and miRNA profiles associated with the 276 miRNA gastric-derived signatures were included in analysis. We also analyzed a signature based on genes differentially expressed in hsa-miR-155-transfected HEK-293 cells (compared to control, nominal p<0.0005 using LIMMA; GSE9264) [39].

### Robustness of miRNA Gene Expression Signatures

To assess the robustness of a miRNA signature, permutation tests were performed where the labels of profiles belonging to the “miRNA high group” and the “miRNA low group” were randomly shuffled 1000 times, and the number of differentially expressed genes between the two permuted groups were counted (permuted signature size). The number of times the permuted signature size exceeded the actual signature size over N = 1000 was taken as the False Discovery Rate (FDR) representing the probability that the signature is not robust (the null hypothesis). A FDR <5% threshold was used in this study to select signatures for further analysis. 276 signatures remained after the signature robustness permutation tests were performed on the original 516 miRNA signatures identified in the discovery set.

### Correlations between miRNA Signature Expression Scores and Actual miRNA Expression Values

miRNA signatures were deemed to be significantly correlated to actual miRNA expression if a signature was both positively correlated to the miRNA (Spearman's correlation R>0) at a threshold q-value of <0.25, where the q-value denotes the significance of the correlation. To compute the q-value of a miRNA signature, we first obtained a nominal p-value representing the significance of the Spearman correlation coefficient between the miRNA signature score and actual miRNA expression. This nominal p-value was then corrected for multiple comparisons using the R function p.adjust, with method option set to “BH” [40], yielding a final q-value. We also used permutation tests to assess the global significance of an observed proportion of miRNA expression signatures positively correlated to actual miRNA expression in the validation data sets. Here, we randomly scrambled the miRNA labels of the miRNA expression signatures 10,000 times, and counted the number of times that percentage of randomized miRNA gene expression scores positively correlated to actual miRNA expression data exceeded the actual data. The value was used as a nominal p-value with which to accept (p>0.05) or reject the null hypothesis (p<0.05), the null hypothesis being that a random miRNA signature is able to predict the expression of a specific miRNA X as accurately as the miRNA X signature itself.

### miRNA–Pathway Network Construction

miRNA and pathway gene expression signatures were mapped onto individual cancer samples using a previously-described signature mapping approach [11], where for each signature (miRNA or pathway) a rank-based, non-parametric method was used to compute a score for each sample representing the ‘activation’ state of that signature. A detailed description of this method is provided in Text S1. Gene expression signatures (n = 174) were obtained from MSigDB (Table S5 provides signature details), and further summarized into 26 major oncogenic pathways reflecting oncogenesis and the tumor microenvironment (see Table S6 for the 26 pathways and the signatures representing them). For the network construction, we only selected those MSigDB C2 signatures representing 26 pathways - This was specifically done to focus our analysis of those pathways relevant to cancer (Akt, Myc, etc), while eliminating from analysis those pathways that were either not related or peripherally related (eg signatures related to heart disease). Significant miRNA–pathway, miRNA-miRNA, and pathway-pathway associations were identified by computing Spearman correlation coefficients (either positive or negative) between the expression/activation scores of each signature pair across the cohort. Because the 26 pathways are summarized from multiple individual signatures, associations involving pathways and a second entity (e.g., a miRNA or another pathway) were only retained when they met the following criteria: at least one signature in the pathway must be significantly positively or negatively correlated with the second entity, and no significantly discordant correlations among the remaining individual signatures are observed. All p-values were corrected for multiple hypothesis testing [41]). Spearman correlations with a corresponding FDR<0.05 were considered. The network associations were iteratively applied to five cancer cohorts: 39 gastric cancer cell lines (“GCCL”, subset of the training gene expression dataset), 200 primary gastric tumors (“SG GC”), 70 primary gastric tumors (“AU GC”), 189 primary breast tumors (“Sotiriou Breast”), and 286 primary breast tumors (“Wang Breast”). Any associations observed to be discordant across any two of the five cohorts were discarded.

### Experimental Validation of Wnt-Related miRNAs

To identify candidate miRNAs regulated by β-catenin knockdown after 24h or 48h β-catenin siRNA treatment, control and β-catenin siRNA treated cells (24h, 48h) were profiled on Human miRNA microarrays (V2) (Agilent). Three independent replicates were compared to identify differentially regulated miRNAs. To measure effects of *hsa-miR-205*, *hsa-miR-221*, *hsa-miR-517c*, and *hsa-miR-519a* on Wnt activity, TOPFLASH assays were conducted on STF-3A cells transfected with these miRNAs as as previously described [42]. All experiments were repeated three independent times. Gene expression profiling using Affymetrix Human Genome U133 plus Genechips (HG-U133 Plus 2.0, Affymetrix) were also performed on pri-hsa-miR-221 and pri-hsa-miR-519a transfected STF-3A cells. Expression profiles were processed using MAS5.0 (R/Bioconductor), log10 transformed, median centered, and subjected to ComBat [43] for batch effect elimination. Geneset Enrichment Analysis (GSEA) was run on the processed dataset using the C2 (curated genesets) subset of MSigDB (Release 2.5). The Wnt-related genesets with the most significant enrichments are discussed, and the complete lists of ranked genesets are provided in Tables S13 and S14.

### Predicted Target mRNAs and Sequence Similarity Analysis

Predicted target mRNAs for the 276 microRNAs analyzed in this study were obtained by combining data from four prediction databases: MiRanda, PicTar, TarScan, and PITA. The final set of predicted target mRNAs for a microRNA is the union of the sets of predicted target mRNAs from all four prediction databases. Here, we chose to use the union rather than the intersect of the various target prediction programs because considering the latter would limit our analysis to only those miRNAs contained in the four databases, which would severely confine subsequent analysis. We used genes (as defined by HUGO symbols) as the basic unit of target mRNAs. Details of the sources for the predicted target mRNAs are available in Table S17. Overlap ratios between the set of target mRNAs for miRNA X, S_X_, and the set of target mRNAs for miRNA Y, S_Y_, were computed as follows: Overlap ratio  =  |(S_X_ ∩ S_Y_)|/min(|S_X_|, |S_Y_|). miRNA sequence information was obtained from ftp://mirbase.org/pub/mirbase/CURRENT/mature.fa.gz and sequence similarity scores between pairs of microRNAs were computed using the *pairwiseAlignment* function from Bioconductor package *Biostrings*. To eliminate microRNA pairs with close sequence similarity, we retained only microRNA pairs with pairwise sequence similarity scores of less than -5. (The sequence similarity score for an identical pair of miRNAs would be 0.)

### Network Models

Random graph networks were generated using the Erdos-Renyi model [44], where all possible pairs of *N* = 302 nodes were connected with probability *p* = 1 and an initial node degree of *k*_initial_ =  44, such that the resulting random graph is equivalent to the miRNA–pathway network in terms of numbers of nodes (302) and mean degree (81.3303±0.2537 for 1000 generated random networks compared to 82.397 for the miRNA–pathway network). Each pairwise correlation was considered as a two-way interaction and no self-correlations were assumed in the microRNA-pathway network. Clustering coefficients for individual nodes *i* were defined as *C_i_*  =  2*n/k_i_*(*k_i_ - 1*), where *n* denotes the number of direct links connecting the *k_i_* nearest neighbors of node *i* [33]. The clustering coefficient for a network *S*, *C_S_*, is the mean of *C_i_* over all nodes *i* in the network *S* and is a measure of the potential modularity of *S*. Hierarchical scale-free networks (HSFN) were generated using a slightly modified version of a previously outlined iterative construction algorithm [45]. Briefly, a 4-level HSFN was first built using a fully connected cluster of 4 nodes as the basic construction unit. Then, a 2-level HSFN was built using a fully connected cluster of 7 nodes as the basic construction unit. The outer nodes of the outer clusters at the second level of the 2-level HSFN were then connected to the central node of the 4-level HSFN, in a manner similar to the second level of the 4-level HSFN itself to that central node. We then eliminated 3 of the outer nodes of the 2-level HSFN so that the final HSFN was exactly of the same size as the microRNA-pathway network (302 nodes). The degree of topological overlap between two nodes i and j, *OT*(*i, j*), was computed as **[** ***#Common neighbors*** **(** ***i, j*** **)]/[min (** ***k_i_, k_j_*** **)]**, where *k_i_, k_j_* are the degrees of node i and node j, respectively.

### Other Statistical Analysis and Visualization Methods

P-values denoting the significance of a corresponding Spearman correlation coefficient R between two N-element vectors were estimated from the Student t-distribution, against the null hypothesis that the observed value of t = R/√[(1−R^2^)/(N–2)] comes from a population in which the true correlation coefficient is zero. Hierarchical clustering and heatmap displays were conducted using BioConductor packages *gplots* and *RColorBrewer*, using default parameters, a Euclidean distance metric and complete linkage.

## Supporting Information

Figure S1 Distribution of Spearman correlation coefficients (R) and q-values (corrected p-values, see Methods) in permuted data. The graphs represent *one* permutation set randomly chosen from 10,000 permutations. The numbers of miRNAs passing the R>0, q<0.25 treshold is shown for the A) gastric, B) breast, C) glioblastoma, and D) ovarian cohorts.
(PDF)

Figure S2 GSEA of miRNA-tranfected cells using gastric-derived miRNA signatures. A) Enrichment plots for the gastric-derived mir-221 signature. (left) Enrichment plot for genes upregulated in the gastric-derived mir-221 signature, queried against genes upregulated in miR-221 transfected cells. (right) Enrichment plot for genes downregulated in the the gastric-derived mir-221 signature, queried against genes downregulated in miR-221 overexpressing cells. B) Enrichment plots for the gastric-derived mir-519a signature (left) Enrichment plot for genes upregulated in the gastric-derived mir-519a signature, queried against genes upregulated in miR-519a transfected cells.upregulated portion of the gastric-derived mir-519a signature in miR-519a overexpressing cells.
(PDF)

Table S1 Gastric-derived miRNA Signatures (Gene Sets).
(RAR)

Table S2 Sizes of gastric-derived miRNA signatures.
(XLS)

Table S3 Statistics comparing permutation tests (n = 10000) vs. actual data for the number of miRNAs with positive correlations between expression scores and expression values (R>0,q<0.25), for all 4 validation sets (GC, OvCA, BC and GBM).
(DOC)

Table S4 Contingency matrices for the null hypothesis that there is no concordance between miRNA expression scores computed from the gastric-derived *hsa-miR-155* signature to miRNA expression scores computed using a HEK-293 cell derived *hsa-miR-155* signature for a) glioblastoma and b) ovarian cancer cohorts.
(DOC)

Table S5 MSigDB signatures analyzed in this study.
(XLS)

Table S6 Expression signatures and pathways.
(XLS)

Table S7 Number of miRNAs correlated to each pathway.
(XLS)

Table S8 Correlations of Spearman correlations (*R*) indicating similarity and reproducibility between sample cohorts for miRNA–pathway associations. *R* = 1 indicates perfect reproducibility. Correlations were computed between pairs of 690×690 matrices. Each matrix represents a sample cohort and contains the correlation coefficients among 174 pathway and 276 miRNA signatures.
(DOC)

Table S9 Predicted miRNA–pathway associations.
(XLS)

Table S10 Top 50 miRNAs targeting multiple pathways.
(XLS)

Table S11 Contingency matrix for Fisher's exact test against the null hypothesis that there is no concordance between previously reported mode of Myc action on the miRNAs (activating or repressing) and the sign of predicted Myc-miRNA correlations (positive or negative). miRNAs positively (negatively) correlated to Myc in the miRNA–pathway network are likely to be previously reported to be Myc-induced (-repressed).
(DOC)

Table S12 Wnt Associated miRNAs: Frequency of Correlations and Regulation by β-catenin.
(XLS)

Table S13 GSEA report for mir-221 transfected STF-3a cells.
(XLS)

Table S14 GSEA report for mir-519a transfected STF-3a cells.
(XLS)

Table S15 Membership of miRNA families used as examples for analysis in Figure 4C.
(DOC)

Table S16 Confusion matrix for chi-square test against the null hypothesis that there is no correlation between miRNA pairs being positively correlated and whether they are from the same family or different families. miRNAs that are from the same family are more likely to be positively correlated than miRNAs from different families.
(DOC)

Table S17 Sources of predicted target mRNAs of the miRNAs.
(DOC)

Table S18 Contingency matrix for chi-square test against the null hypothesis that there is no correlation between miRNA pairs being positively correlated and whether the overlap ratio of their common targets is large (>0.6). miRNAs that are positively correlated to each other are more likely to have large overlap of target mRNAs (overlap ratio greater than 0.6) compared to miRNAs that are not positively correlated to each other. miRNAs with large overlaps of target mRNAs (overlap ratio greater than 0.6) are more likely to be positively correlated than miRNAs with small overlap of target mRNAs.
(DOC)

Table S19 Contingency matrix for chi-square test against the null hypothesis that miRNA–pathway interactions are not associated with the genomic cluster of the miRNAs. miRNA pairs transcribed from a common genomic cluster are twice as likely to co-interact with at least one common pathway as miRNA pairs transcribed from different clusters.
(DOC)

Table S20 Confusion matrix for pathway cotargeting. (A) Chi-square test against the null hypothesis that miRNA–pathway interactions are not associated with the number of predicted mRNA targets. miRNA pairs with large overlaps in predicted target mRNAs are twice as likely to co-interact with at least one common pathway as miRNA pairs with small overlaps in targets. (B) Previous analysis repeated but retaining only miRNA pairs with pairwise sequence similarity score of less than -5, to remove sequence similarity as a confounding factor.
(DOC)

Text S1 Computation of pathway activation scores.
(PDF)
