# Supplementary material for: A Densely Interconnected Genome-Wide Network of MicroRNAs and Oncogenic Pathways Revealed Using Gene Expression Signatures
Source: PLoS Genet. 2011 Dec 15;7(12):e1002415. doi: 10.1371/journal.pgen.1002415 (PMC3240594; doi:10.1371/journal.pgen.1002415)

Figure S1. Distribution of Spearman correlation coefficients (R) and q-values (corrected p-values, see Methods) in permuted data. The graphs represent *one* permutation set randomly chosen from 10,000 permutations. The numbers of miRNAs passing the R>0, q<0.25 treshold is shown for the A) gastric, B) breast, C) glioblastoma, and D) ovarian cohorts.

A **A random permutation of 31 primary gastric tumors**

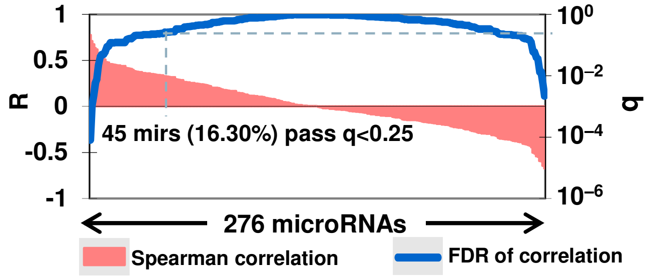

B **A random permutation of 578 ovarian serous cystadenocarcinomas**

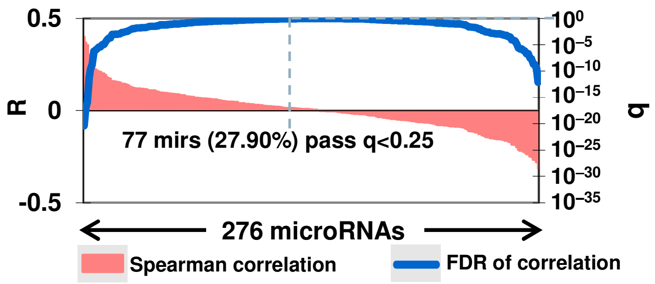

C **A random permutation of 99 primary breast tumors**

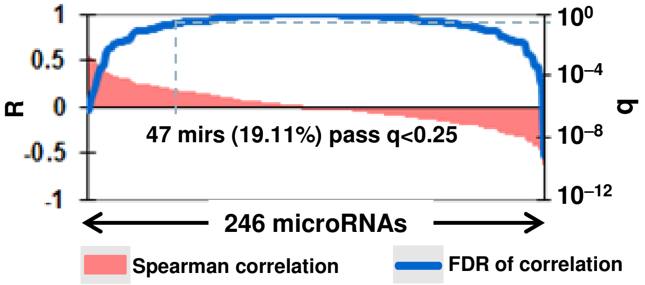

D **A random permutation of 418 glioblastoma multiformes**

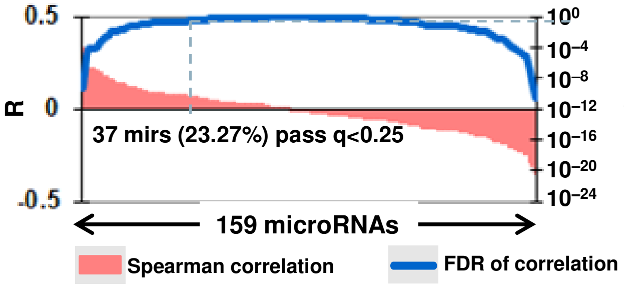

Supplement: Figure S1 — Distribution of Spearman correlation coefficients (R) and q-values (corrected p-values, see Methods) in permuted data. The graphs represent one permutation set randomly chosen from 10,000 permutations. The numbers of miRNAs passing the R>0, q<0.25 treshold is shown for the A) gastric, B) breast, C) glioblastoma, and D) ovarian cohorts. (PDF) [file pgen.1002415.s001.pdf]
