# Supplementary material for: A Densely Interconnected Genome-Wide Network of MicroRNAs and Oncogenic Pathways Revealed Using Gene Expression Signatures
Source: PLoS Genet. 2011 Dec 15;7(12):e1002415. doi: 10.1371/journal.pgen.1002415 (PMC3240594; doi:10.1371/journal.pgen.1002415)
Supplement: Figure S2 — GSEA of miRNA-tranfected cells using gastric-derived miRNA signatures. A) Enrichment plots for the gastric-derived mir-221 signature. (left) Enrichment plot for genes upregulated in the gastric-derived mir-221 signature, queried against genes upregulated in miR-221 transfected cells. (right) Enrichment plot for genes downregulated in the the gastric-derived mir-221 signature, queried against genes downregulated in miR-221 overexpressing cells. B) Enrichment plots for the gastric-derived mir-519a signature (left) Enrichment plot for genes upregulated in the gastric-derived mir-519a signature, queried against genes upregulated in miR-519a transfected cells.upregulated portion of the gastric-derived mir-519a signature in miR-519a overexpressing cells. (PDF) [file pgen.1002415.s002.pdf]

Figure S2. GSEA of miRNA-tranfected cells using gastric-derived miRNA signatures.

A) Enrichment plots for the gastric-derived mir-221 signature. (left) Enrichment plot for genes upregulated in the gastric-derived mir-221 signature, queried against genes upregulated in miR-221 transfected cells. (right) Enrichment plot for genes downregulated in the the gastric-derived mir-221 signature, queried against genes downregulated in miR-221 overexpressing cells.

B) Enrichment plots for the gastric-derived mir-519a signature (left) Enrichment plot for genes upregulated in the gastric-derived mir-519a signature, queried against genes upregulated in miR-519a transfected cells.upregulated portion of the gastric-derived mir-519a signature in miR-519a overexpressing cells.

A, left panel:
Nominal p-value =0.66721314
FDR q-value= 0.68419695

A, right panel:
Nominal p-value =0.019736841
FDR q-value= 0.047513805

B, left panel:
Nominal p-value=0.3548387
FDR q-value= 0.70931417

A

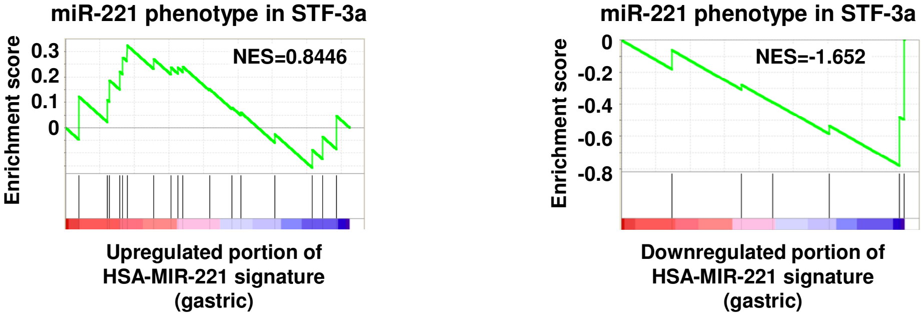

B

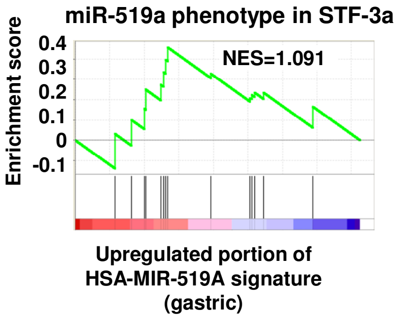

There is only one gene (Affy probeset ID) in the downregulated portion of HSA-MIR-519A signature (gastric), so it was not included in the GSEA.
