# Supplementary material for: A Densely Interconnected Genome-Wide Network of MicroRNAs and Oncogenic Pathways Revealed Using Gene Expression Signatures
Source: PLoS Genet. 2011 Dec 15;7(12):e1002415. doi: 10.1371/journal.pgen.1002415 (PMC3240594; doi:10.1371/journal.pgen.1002415)
Supplement: Table S3 — Statistics comparing permutation tests (n = 10000) vs. actual data for the number of miRNAs with positive correlations between expression scores and expression values (R>0,q<0.25), for all 4 validation sets (GC, OvCA, BC and GBM). (DOC) [file pgen.1002415.s005.doc]

**Table S3.** Statistics comparing permutation tests (n=10000) vs. actual data for the number of miRNAs with positive correlations between expression scores and expression values (R>0,q<0.25), for all 4 validation sets (GC, OvCA, BC and GBM).

| Validation dataset | **Actual # miRNA signatures passing q<0.25 and R>0 threshold in actual data** | Average # permuted miRNA sigantures passing q<0.25 and R>0 threshold across 10000 permutations (per data set) | Maximum # of permuted miRNA signatures passing q<0.25 and R>0 threshold across all 10000 permutations |
| --- | --- | --- | --- |
| GC | **101** | 42.4176 | 71 |
| SCA (Ovarian) | **171** | 71.6188 | 101 |
| BC | **94** | 46.2994 | 72 |
| GBM | **53** | 37.2232 | 61 |
