# Supplementary material for: A Densely Interconnected Genome-Wide Network of MicroRNAs and Oncogenic Pathways Revealed Using Gene Expression Signatures
Source: PLoS Genet. 2011 Dec 15;7(12):e1002415. doi: 10.1371/journal.pgen.1002415 (PMC3240594; doi:10.1371/journal.pgen.1002415)
Supplement: Table S4 — Contingency matrices for the null hypothesis that there is no concordance between miRNA expression scores computed from the gastric-derived hsa-miR-155 signature to miRNA expression scores computed using a HEK-293 cell derived hsa-miR-155 signature for a) glioblastoma and b) ovarian cancer cohorts. (DOC) [file pgen.1002415.s006.doc]

**Table S4.** Contingency matrices for the null hypothesis that there is no concordance between miRNA expression scores computed from the gastric-derived *hsa-miR-155* signature to miRNA expression scores computed using a HEK-293 cell derived *hsa-miR-155* signature for a) glioblastoma and b) ovarian cancer cohorts.


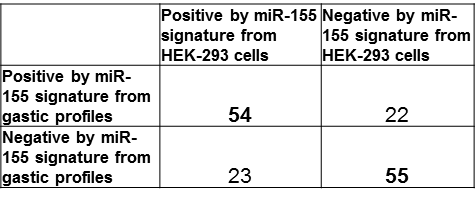
A)

**p=5.8373E-07 (chi-square test)**


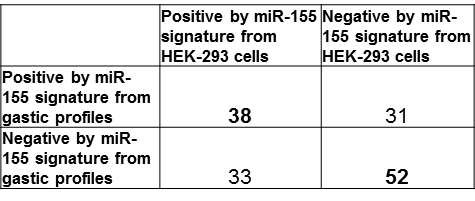
B)

**p=0.0644 (chi-square test)**
