# Supplementary material for: A Densely Interconnected Genome-Wide Network of MicroRNAs and Oncogenic Pathways Revealed Using Gene Expression Signatures
Source: PLoS Genet. 2011 Dec 15;7(12):e1002415. doi: 10.1371/journal.pgen.1002415 (PMC3240594; doi:10.1371/journal.pgen.1002415)
Supplement: Table S8 — Correlations of Spearman correlations (R) indicating similarity and reproducibility between sample cohorts for miRNA–pathway associations. R = 1 indicates perfect reproducibility. Correlations were computed between pairs of 690×690 matrices. Each matrix represents a sample cohort and contains the correlation coefficients among 174 pathway and 276 miRNA signatures. (DOC) [file pgen.1002415.s010.doc]

**Table S8.** **Correlations of Spearman correlations (*R*) indicating similarity and reproducibility between sample cohorts for miRNA-pathway associations.** *R*=1 indicates perfect reproducibility. Correlations were computed between pairs of 690x690 matrices. Each matrix represents a sample cohort and contains the correlation coefficients among 174 pathway and 276 miRNA signatures.

| ***R*** | **GEMINI GC** | **SG GC** | **AU GC** | **Sotiriou Breast** | **Wang Breast** |
| --- | --- | --- | --- | --- | --- |
| **GEMINI GC** | 0.000 | 0.399 | 0.384 | 0.232 | 0.256 |
| **SG GC** | 0.399 | 0.000 | 0.674 | 0.430 | 0.447 |
| **AU GC** | 0.384 | 0.674 | 0.000 | 0.387 | 0.406 |
| **Sotiriou Breast** | 0.232 | 0.430 | 0.387 | 0.000 | 0.690 |
| **Wang Breast** | 0.256 | 0.447 | 0.406 | 0.690 | 0.000 |

Green boxes: correlations between cohorts of similar tissue type.

Red boxes: correlations between cohorts of different tissue type

Results : All correlations are positive with associated p-values < 1x10^-6^, indicating that the miRNA-pathway associations are reproducible across different cancer cohorts.

There is no significant difference between same tissue type correlations (e.g., gastric vs. gastric or breast vs. breast) and different tissue type correlations (gastric vs. breast) (p=0.2864, Kruskal-Wallis test). The lack of significance persists even after exckuding the GEMINI GC cohort (p=0.0641, Kruskal-Wallis test).

These results suggest that a many of the miRNA-pathway associations are preserved even in tumors of different tissue types. However, we emphasize that this result does not rule out that certain miRNAs may also exert tissue-specific effects (see Discussion in Main Text).
