# Supplementary material for: A Densely Interconnected Genome-Wide Network of MicroRNAs and Oncogenic Pathways Revealed Using Gene Expression Signatures
Source: PLoS Genet. 2011 Dec 15;7(12):e1002415. doi: 10.1371/journal.pgen.1002415 (PMC3240594; doi:10.1371/journal.pgen.1002415)
Supplement: Table S11 — Contingency matrix for Fisher's exact test against the null hypothesis that there is no concordance between previously reported mode of Myc action on the miRNAs (activating or repressing) and the sign of predicted Myc-miRNA correlations (positive or negative). miRNAs positively (negatively) correlated to Myc in the miRNA–pathway network are likely to be previously reported to be Myc-induced (-repressed). (DOC) [file pgen.1002415.s013.doc]

**Table S11.** Contingency matrix for Fisher’s exact test against the null hypothesis that there is no concordance between previously reported mode of Myc action on the miRNAs (activating or repressing) and the sign of predicted Myc-miRNA correlations (positive or negative). miRNAs positively (negatively) correlated to Myc in the miRNA-pathway network are likely to be previously reported to be Myc-induced (-repressed).

| **Observed** | **MicroRNA is reported as Myc-induced** | **MicroRNA is reported as Myc-repressed** |  |
| --- | --- | --- | --- |
| MicroRNA is positively correlated to Myc in at least 1 cohort | **10** | 1 | 11 |
| MicroRNA is negatively correlated to Myc in at least 1 cohort | 0 | **7** | 7 |
|  | 10 | 8 | **18** |
| **p=0.000251383** |  |  |  |
|  |  |  |  |
