# Supplementary material for: A Densely Interconnected Genome-Wide Network of MicroRNAs and Oncogenic Pathways Revealed Using Gene Expression Signatures
Source: PLoS Genet. 2011 Dec 15;7(12):e1002415. doi: 10.1371/journal.pgen.1002415 (PMC3240594; doi:10.1371/journal.pgen.1002415)
Supplement: Table S15 — Membership of miRNA families used as examples for analysis in Figure 4C. (DOC) [file pgen.1002415.s017.doc]

**Table S15.** Membership of miRNA families used as examples for analysis in Figure 4C.

| **Family** | **MicroRNA members of the family** |
| --- | --- |
| miR-200 family | hsa-miR-141/*, hsa-miR-200a/a*/b/b*/c/c*, hsa-miR-429 |
| mir-17 family | hsa-miR-106b, hsa-miR-17/*, hsa-miR-18a/b, hsa-miR-20a/*, hsa-miR-93 |
| miR-30 family | hsa-miR-30a/a*/c/d/e*, hsa-miR-30c-2* |
| miR-10 family | hsa-miR-100/*, hsa-miR-10b, hsa-miR-99a/a*/b |
