# Supplementary material for: A Densely Interconnected Genome-Wide Network of MicroRNAs and Oncogenic Pathways Revealed Using Gene Expression Signatures
Source: PLoS Genet. 2011 Dec 15;7(12):e1002415. doi: 10.1371/journal.pgen.1002415 (PMC3240594; doi:10.1371/journal.pgen.1002415)
Supplement: Table S16 — Confusion matrix for chi-square test against the null hypothesis that there is no correlation between miRNA pairs being positively correlated and whether they are from the same family or different families. miRNAs that are from the same family are more likely to be positively correlated than miRNAs from different families. (DOC) [file pgen.1002415.s018.doc]

**Table S16.** Confusion matrix for chi-square test against the null hypothesis that there is no correlation between miRNA pairs being positively correlated and whether they are from the same family or different families. miRNAs that are from the same family are more likely to be positively correlated than miRNAs from different families.

| **Observed** | **Same family** | **Different families** |  |
| --- | --- | --- | --- |
| MiRNA pair is positively correlated | **63** | 34 | 97 |
| MiRNA pair is not positively correlated | 31 | **278** | 309 |
|  | 94 | 312 | **406** |
| **p=4.75872E-29** |  |  |  |
|  |  |  |  |
