# Supplementary material for: A Densely Interconnected Genome-Wide Network of MicroRNAs and Oncogenic Pathways Revealed Using Gene Expression Signatures
Source: PLoS Genet. 2011 Dec 15;7(12):e1002415. doi: 10.1371/journal.pgen.1002415 (PMC3240594; doi:10.1371/journal.pgen.1002415)
Supplement: Table S17 — Sources of predicted target mRNAs of the miRNAs. (DOC) [file pgen.1002415.s019.doc]

**Table S17. Sources of predicted target mRNAs of the miRNAs.**

| **Algorithm** | **Source** |
| --- | --- |
| MiRanda | <http://cbio.mskcc.org/microrna_data/human_predictions_sept2008.txt.gz> |
| PicTar | http://www.ncrna.org/glocal/cgi-bin/hgTables?hgsid=2&clade=mammal&org=Human&db=hg18&hgta_group=miRNA&hgta_track=pred_Target&hgta_table=picTarMiRNA4Way&hgta_regionType=range&position=chrX%3A151073054-151383976&hgta_outputType=primaryTable&hgta_outFileName=yuzeqsmofb |
| TarScan | http://www.targetscan.org//vert_50//vert_50_data_download/Predicted_Targets_Info.txt.zip |
| PITA | http://www.ncrna.org/glocal/cgi-bin/hgTables?hgsid=2&clade=mammal&org=Human&db=hg18&hgta_group=miRNA&hgta_track=pred_Target&hgta_table=PITA_top&hgta_regionType=genome&position=chrX%3A151073054-151383976&hgta_outputType=primaryTable&hgta_outFileName=yuzeqsmofb |
