# Supplementary material for: A Densely Interconnected Genome-Wide Network of MicroRNAs and Oncogenic Pathways Revealed Using Gene Expression Signatures
Source: PLoS Genet. 2011 Dec 15;7(12):e1002415. doi: 10.1371/journal.pgen.1002415 (PMC3240594; doi:10.1371/journal.pgen.1002415)
Supplement: Table S18 — Contingency matrix for chi-square test against the null hypothesis that there is no correlation between miRNA pairs being positively correlated and whether the overlap ratio of their common targets is large (>0.6). miRNAs that are positively correlated to each other are more likely to have large overlap of target mRNAs (overlap ratio greater than 0.6) compared to miRNAs that are not positively correlated to each other. miRNAs with large overlaps of target mRNAs (overlap ratio greater than 0.6) are more likely to be positively correlated than miRNAs with small overlap of target mRNAs. (DOC) [file pgen.1002415.s020.doc]

**Table S18.** Contingency matrix for chi-square test against the null hypothesis that there is no correlation between miRNA pairs being positively correlated and whether the overlap ratio of their common targets is large (> 0.6). miRNAs that are positively correlated to each other are more likely to have large overlap of target mRNAs (overlap ratio greater than 0.6) compared to miRNAs that are not positively correlated to each other. miRNAs with large overlaps of target mRNAs (overlap ratio greater than 0.6) are more likely to be positively correlated than miRNAs with small overlap of target mRNAs.

| **Observed** | **Overlap ratio between target mRNAs of miRNA pair > 0.6** | **Overlap ratio between target mRNAs of miRNA pair < 0.4** |  |
| --- | --- | --- | --- |
| MiRNA pair is positively correlated | **36** | 4463 | 4499 |
| MiRNA pair is not positively correlated | 28 | **18225** | 18253 |
|  | 64 | 22688 | **22752** |
| **p=2.18882E-13** |  |  |  |
|  |  |  |  |
