# Supplementary material for: A Densely Interconnected Genome-Wide Network of MicroRNAs and Oncogenic Pathways Revealed Using Gene Expression Signatures
Source: PLoS Genet. 2011 Dec 15;7(12):e1002415. doi: 10.1371/journal.pgen.1002415 (PMC3240594; doi:10.1371/journal.pgen.1002415)
Supplement: Table S19 — Contingency matrix for chi-square test against the null hypothesis that miRNA–pathway interactions are not associated with the genomic cluster of the miRNAs. miRNA pairs transcribed from a common genomic cluster are twice as likely to co-interact with at least one common pathway as miRNA pairs transcribed from different clusters. (DOC) [file pgen.1002415.s021.doc]

**Table S19.** Contingency matrix for chi-square test against the null hypothesis that miRNA-pathway interactions are not associated with the genomic cluster of the miRNAs. miRNA pairs transcribed from a common genomic cluster are twice as likely to co-interact with at least one common pathway as miRNA pairs transcribed from different clusters.

| **Observed** | **MiRNA pair belongs to the same genomic cluster** | **MiRNA pair belongs to different genomic clusters** |  |
| --- | --- | --- | --- |
| MiRNA pair is never connected to the same pathway | 8 | **319** | 327 |
| MiRNA pair is connected to same pathway at least once | **34** | 200 | 234 |
|  | 42 | 519 | **561** |
| **p=8.22022E-08** |  |  |  |
|  |  |  |  |
