# Supplementary material for: A Densely Interconnected Genome-Wide Network of MicroRNAs and Oncogenic Pathways Revealed Using Gene Expression Signatures
Source: PLoS Genet. 2011 Dec 15;7(12):e1002415. doi: 10.1371/journal.pgen.1002415 (PMC3240594; doi:10.1371/journal.pgen.1002415)
Supplement: Table S20 — Confusion matrix for pathway cotargeting. (A) Chi-square test against the null hypothesis that miRNA–pathway interactions are not associated with the number of predicted mRNA targets. miRNA pairs with large overlaps in predicted target mRNAs are twice as likely to co-interact with at least one common pathway as miRNA pairs with small overlaps in targets. (B) Previous analysis repeated but retaining only miRNA pairs with pairwise sequence similarity score of less than -5, to remove sequence similarity as a confounding factor. (DOC) [file pgen.1002415.s022.doc]

**Table S20a. Confusion matrix for chi-square test against the null hypothesis that miRNA-pathway interactions are not associated with the number of predicted mRNA targets. miRNA pairs with large overlaps in predicted target mRNAs are twice as likely to co-interact with at least one common pathway as miRNA pairs with small overlaps in targets.**

| **Observed** | **Overlap ratio between target mRNAs of miRNA pair > 0.6** | **Overlap ratio between target mRNAs of miRNA pair < 0.4** |  |
| --- | --- | --- | --- |
| MiRNA pair is never connected to the same pathway | 17 | **14728** | 14745 |
| MiRNA pair is connected to same pathway at least once | **47** | 7960 | 8007 |
|  | 64 | 22688 | **22752** |
| **p=1.40253E-10** |  |  |  |
|  |  |  |  |

**Table S20b.** Previous analysis repeated but by retaining only miRNA pairs with pairwise sequence similarity score of less than -5, to remove sequence similarity as a confounding factor.

| **Observed** | **Overlap ratio between target mRNAs of miRNA pair > 0.6** | **Overlap ratio between target mRNAs of miRNA pair < 0.4** |  |
| --- | --- | --- | --- |
| MiRNA pair is never connected to the same pathway | 9 | **14199** | 14208 |
| MiRNA pair is connected to same pathway at least once | **19** | 7828 | 7847 |
|  | 28 | 22027 | **22055** |
| **p=0.000357196** |  |  |  |
|  |  |  |  |
