# Supplementary material for: A Densely Interconnected Genome-Wide Network of MicroRNAs and Oncogenic Pathways Revealed Using Gene Expression Signatures
Source: PLoS Genet. 2011 Dec 15;7(12):e1002415. doi: 10.1371/journal.pgen.1002415 (PMC3240594; doi:10.1371/journal.pgen.1002415)
Supplement: Text S1 — Computation of pathway activation scores. (PDF) [file pgen.1002415.s023.pdf]

Text S1 : Computation of Pathway Activation Scores

Pathway activation scores were computed as described in [1]. This methodology uses two inputs : 1) cancer profiles, comprising lists of probesets sorted by differential gene expression between individual cancer gene expression profiles and a reference profile (see Section *Reference Profiles*), where n is defined as the total number of probesets in each cancer profile i, and 2) a query signature QS (pathway activation signature). The sorting procedure used to generate the cancer profiles was described in the Supplementary Info of a previous study [2] A similar strategy was also used to compute miRNA expression scores across the samples.

For the QS, probesets representing either up- (or down-) regulated genes in the QS are defined as 'tags', and t the number of tags in the up- (or down-) regulated portion of the QS. Raw enrichment scores $k^i_{\text{direction}}$ were computed using a Kolmogorov-Smirnov metric previously described in [2]. Here, 'direction' in $k^i_{\text{direction}}$ may be considered as 'up' or 'down', depending on whether the set of tags in question represents the up-regulated (k^i_{up}) or the down-regulated (k^i_{down}) portion of the QS. For a cancer profile i and a set of t QS tags, the position of tag j in the cancer profile i is defined as $V(j)$, forming the vector **V.**

$$\mathbf{V} = [V(1) \quad V(2) \quad \ldots \quad V(t)] \qquad \text{[a]}$$

The elements of **V** are then sorted in ascending order of $V(j)$ such that $V(1) \le V(2) \le \ldots \le V(t-1) \le V(t)$. In this manner, the tags indexed by j are ordered based on their position in the cancer profile (e.g. tag 1 is the probeset with the highest rank in the cancer profile among all t tags in the up- (or down-) regulated portion of the QS). Using the sorted elements of **V**, two parameters are computed:

$$a = \max_{j=1}^{t}\left[\frac{j}{t} - \frac{V(j)}{n}\right] \qquad \text{[b]}$$

$$b = \max_{j=1}^{t}\left[\frac{V(j)}{n} - \frac{(j-1)}{t}\right] \qquad \text{[c]}$$

If $a > b$, $k^i_{\text{direction}}$ is set to a. Otherwise, (if $b > a$), $k^i_{\text{direction}}$ is set to $-b$.

To compute the pathway activation score S^i, if k^i_{up} and k^i_{down} have the same signs then S^i for cancer profile i is set to zero. Otherwise, the raw activation score s^i is obtained.

$$s^i = k^i_{\text{up}} - k^i_{\text{down}} \quad [d]$$

The maximum and minimum of s^i across all cancer profiles in the cohort are defined as p and q, respectively. The activation score S^i is the normalized form of s^i, where

$$S^i = \frac{s^i}{p} \qquad \text{if} \quad s^i > 0 \quad [e]$$

and

$$S^i = -\frac{s^i}{q} \qquad \text{if} \quad s^i < 0 \quad [f]$$

In cases where more than one profile exists for a sample, the final activation score represents the mean activation score across the replicate profiles.

Reference Profiles. For all cohorts analysed in this study except for the gastric cell line cohorts, activation scores were computed using the median profile of the cohort as the reference profile. The median profile was obtained by computing the median of expression values across all members of the cohort. For gastric cell line profiles, we used seven distinct reference profiles: the median gastric cell line profile, a normal skin fibroblast profile, and five normal stomach profiles (see Table below). Final activation scores for the gastric cell line profiles were obtained by computing the mean activation scores across the seven reference profiles.

Reference profiles for gastric cell lines.

No.	Reference name	Details	#Replicates or profiles	GEO Accession #
1	Median GCCL	Median of GC cell lines	1	GSE15455
2	CRL2072	Normal skin fibroblasts, 3 replicates	3	GSE15455
3	STN_CARDIAC	Stomach cardiac, 2 male and 1 female profiles	3	GSE7307
4	STN_PYLORIC	Stomach pyloric, 3 male and 1 female profiles	4	GSE7307
5	STN_FUNDUS	Stomach fundus, 3 male and 1 female profiles	4	GSE7307
6	STN_BD	Normal stomach, 1 profile from M/F mix	1	GSE7307
7	STN_GSE7307	Combination of 3-6	12	GSE7307

References

1. Ooi CH, Ivanova T, Wu J, Lee M, Tan IB, et al. (2009) Oncogenic pathway combinations predict clinical prognosis in gastric cancer. PLoS Genet 5: 10.
2. Lamb J, Crawford ED, Peck D, Modell JW, Blat IC, et al. (2006) The Connectivity Map: using gene-expression signatures to connect small molecules, genes, and disease. Science 313(5795): 1929 - 1935.
